# Supplementary material for: Seasonality and climate modes influence the temporal clustering of unique atmospheric rivers in the Western U.S
Source: Commun Earth Environ. 2024 Nov 23;5(1):734. doi: 10.1038/s43247-024-01890-x (PMC11584389; doi:10.1038/s43247-024-01890-x)
Supplement: Supplementary file 2 — Supplementary Information [file 43247_2024_1890_MOESM2_ESM.pdf]

Seasonality and climate modes influence the temporal clustering of unique atmospheric rivers in the Western U.S.

Zhiqi Yang*[1], Michael J. DeFlorio[1], Agniv Sengupta[1], Jiabao Wang[1], Christopher M. Castellano[1], Alexander Gershunov[1], Kristen Guirguis[1], Emily Slinskey[1], Bin Guan[2,3], Luca Delle Monache[1], F. Martin Ralph[1]

[1]Center for Western Weather and Water Extremes, Scripps Institution of Oceanography, University of California San Diego, San Diego, CA, USA

[2]Joint Institute for Regional Earth System Science and Engineering, University of California Los Angeles, Los Angeles, CA, USA

[3]Jet Propulsion Laboratory, California Institute of Technology, Pasadena, CA, USA

Corresponding author address:

Zhiqi Yang, zhy040@ucsd.edu. Center for Western Weather and Water Extremes, Scripps Institution of Oceanography, University of California San Diego, San Diego, CA, USA.

Supplementary:

Supplementary Figure 1

Supplementary Figure 2

Supplementary Figure 3

Supplementary Figure 4

Supplementary Table 1

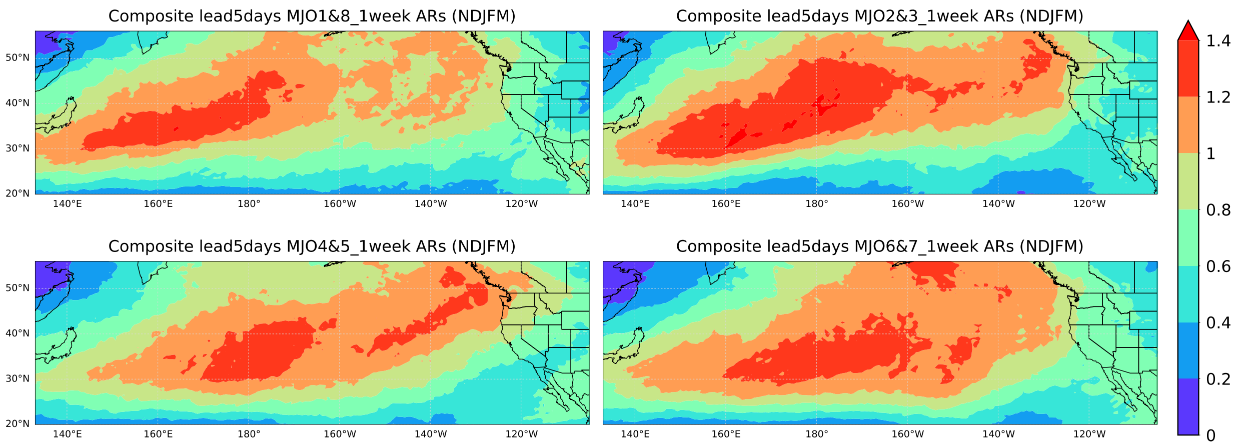

Supplementary Figure 1. Composite analysis of the number of unique ARs in a 1-week window during extended winter (NDJFM) based on four combined MJO phases using the MERRA-2 AR dataset from 1982/1983 to 2020/2021. Unit: number of unique ARs.

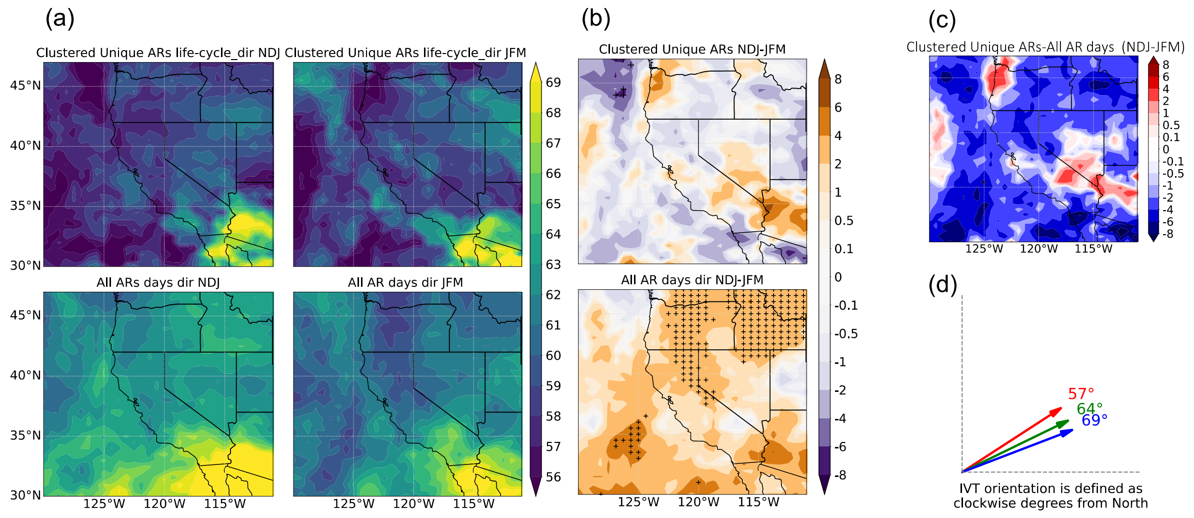

Supplementary Figure 2. **Panel (a):** Life-cycle IVT orientation of temporal clustered unique ARs in early winter (NDJ, top left) and late winter (JFM, top right). IVT orientation of all AR days in early winter (NDJ, bottom left) and late winter (JFM, bottom right). Data derived from the MERRA-2 AR dataset from 1982/1983 to 2020/2021. Unit: degree. **Panel (b):** Life-cycle IVT orientation of temporal clustered unique ARs for NDJ minus JFM (top). IVT orientation of all AR days for NDJ minus JFM (bottom). Dots indicate statistical significance at the 5% level. Unit: degree. **Panel (c):** Panel (b) top minus bottom. **Panel (d):** IVT orientation diagram.

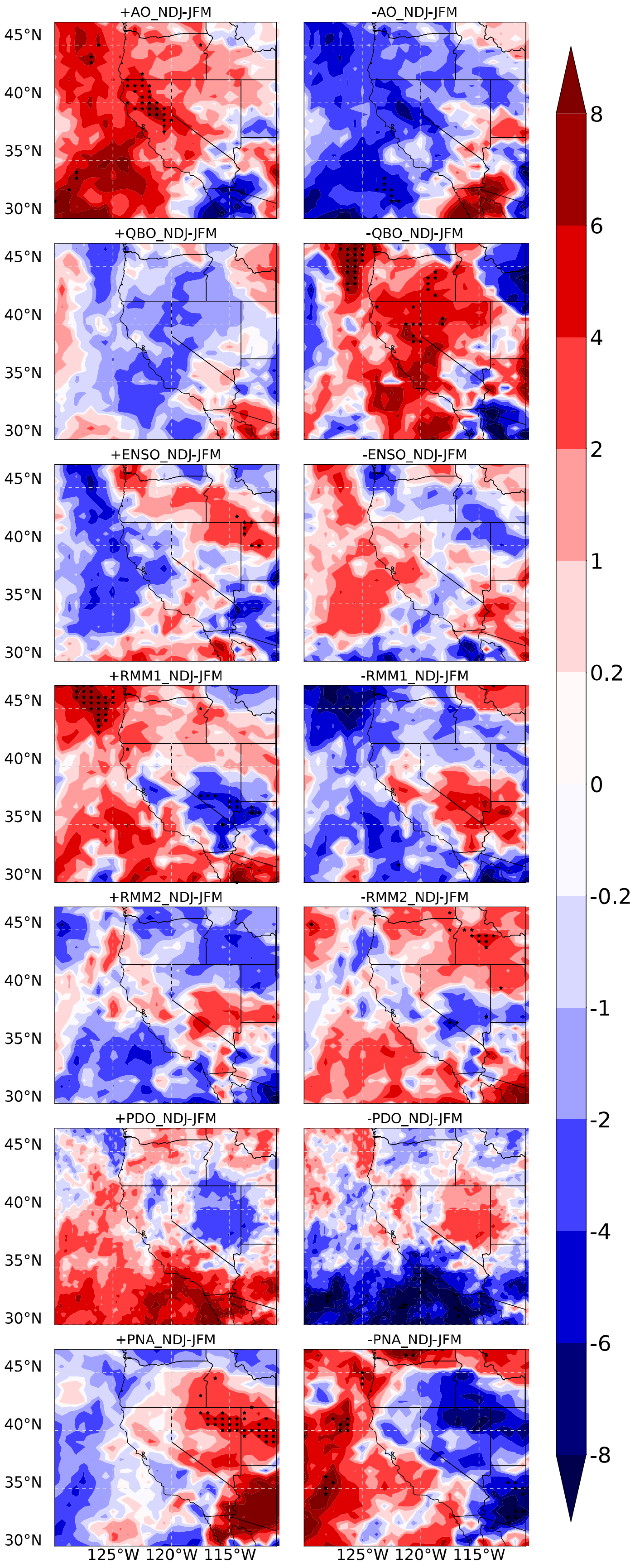
+AO_NDJ-JFM
-AO_NDJ-JFM
+QBO_NDJ-JFM
-QBO_NDJ-JFM
+ENSO_NDJ-JFM
-ENSO_NDJ-JFM
+RMM1_NDJ-JFM
-RMM1_NDJ-JFM
+RMM2_NDJ-JFM
-RMM2_NDJ-JFM
+PDO_NDJ-JFM
-PDO_NDJ-JFM
+PNA_NDJ-JFM
-PNA_NDJ-JFM
45°N
40°N
35°N
30°N
125°W 120°W 115°W
8
6
4
2
1
0.2
0
-0.2
-1
-2
-4
-6
-8

Supplementary Figure 3. Composite analysis in anomalies of the life-cycle IVT orientation of temporally clustered unique AR of early winter (NDJ) minus late winter (JFM). The analysis based on positive and negative phases of ENSO, QBO, MJO-RMM1, MJO-RMM2, PNA (removed ENSO signal), AO, PDO, using the ERA-5 AR dataset from 1940/1941 to 2017/2018 (for PDO analysis) and the MERRA-2 AR dataset from 1982/1983 to 2020/2021 (for other climate modes analysis). Dots indicate statistically significant at the 5% level. Unit: degree.

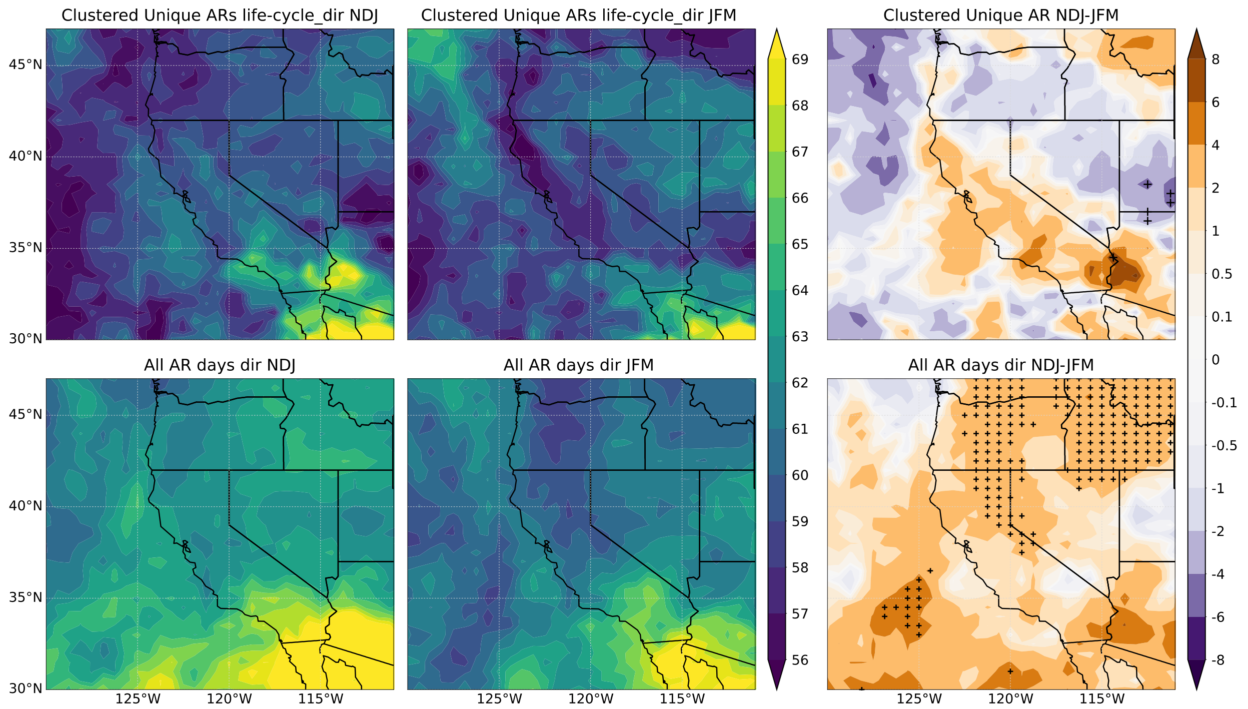

Supplementary Figure 4. Sensitive tests by removing the first 2 days from the analysis in Figure S2(a)(b) (also in Figure 9(b) Life-cycle IVT orientation of temporal clustered unique ARs) and then recalculating the clustering for both NDJ and JFM periods. Life-cycle IVT orientation of temporal clustered unique ARs in early winter (NDJ, top left), late winter (JFM, top middle), and their difference (top right). IVT orientation of all AR days in early winter (NDJ, bottom left), late winter (JFM, bottom middle), and their difference (bottom right). Data derived from the MERRA-2 AR dataset from 1982/1983 to 2020/2021. Dots indicate statistically significant at the 5% level. Unit: degree.

Supplementary Table 1. The correlation coefficients of all pairs of AO, PNA (after removing ENSO), MJO, QBO, and ENSO:

**Climate Modes**	**NDJ**	**JFM**	**NDJFM**
**AO, PNA**	-0.2001486018273725	-0.24637864819105046	-0.22586641135570698
**AO, RMM1**	0.1209974433901579	0.13668287155888773	0.12040836802640792
**AO, RMM2**	-0.14273152700488279	-0.09281873451330258	-0.11016684247253844
**AO, QBO**	0.09406993774545983	-0.002884660187138659	0.04219392930520683
**AO, ENSO**	0.04970780835261645	-0.05224780847040277	0.017161228297032597
**PNA, RMM1**	-0.12115710713056552	-0.21255015548629774	-0.14734450058746087
**PNA, RMM2**	0.011772280426781672	0.06678823179269228	0.06126415754558458
**PNA, QBO**	-0.004741184607769526	0.04540358271568377	0.04404277598393147
**PNA, ENSO**	$-6.121725906730966\times10^{-5}$	0.0012393480442218633	0.07669100607194652
**RMM1, RMM2**	-0.026638671121564098	-0.10424254972073081	-0.08375252047633747
**RMM1, QBO**	0.06267201328652588	0.0036516165803568298	0.018184691699926075
**RMM1, ENSO**	0.028306267340458997	-0.011848280923704407	0.0160019803792233
**RMM2, QBO**	-0.05533665225368805	-0.08861361561655196	-0.06668185623789367
**RMM2, ENSO**	-0.13531087401849876	-0.01258077309207827	-0.10997883961040632
**QBO, ENSO**	0.18525855802209815	0.14935683005025127	0.15679703260914
